# Racial Disparities in Patient Characteristics and Survival After Acute Myocardial Infarction

**DOI:** 10.1001/jamanetworkopen.2018.4240

**Published:** 2018-11-02

**Authors:** Garth N. Graham, Philip G. Jones, Paul S. Chan, Suzanne V. Arnold, Harlan M. Krumholz, John A. Spertus

**Affiliations:** 1 Department of Cardiovascular Research, Saint Luke’s Mid America Heart Institute, Kansas City, Missouri; 2 University of Missouri–Kansas City School of Medicine, Kansas City; 3 Center for Outcomes Research and Evaluation, Yale New Haven Hospital, New Haven, Connecticut; 4 Section of Cardiovascular Medicine, Department of Medicine, Yale School of Medicine, New Haven, Connecticut

## Abstract

**Importance:**

Black patients experience worse outcomes than white patients following acute myocardial infarction (AMI).

**Objective:**

To examine the degree to which nonrace characteristics explain observed survival differences between white patients and black patients following AMI.

**Design, Setting, and Participants:**

This cohort study used the extensive socioeconomic and clinical characteristics from patients recovering from an AMI that were prospectively collected at 31 hospitals across the contiguous United States between 2003 and 2008 for the Prospective Registry Evaluating Myocardial Infarction: Events and Recovery registry and the Translational Research Investigating Underlying Disparities in Acute Myocardial Infarction Patients’ Health Status registry. Survival was assessed using data from the National Death Index. Data were analyzed from December 2016 to July 2018.

**Main Outcomes and Measures:**

Patient characteristics were categorized into 8 domains, and the degree to which each domain discriminated self-identified black patients from white patients was determined by calculating propensity scores associated with black race for each domain as well as cumulatively across all domains. The final propensity score was associated with 1- and 5-year mortality rates.

**Results:**

Among 6402 patients (mean [SD] age, 60 [13] years; 2127 [33.2%] female; 1648 [25.7%] black individuals), the 5-year mortality rate following AMI was 28.9% (476 of 1648) for black patients and 18.0% (856 of 4754) for white patients (hazard ratio, 1.72; 95% CI, 1.54-1.92; *P* < .001). Most categories of patient characteristics differed substantially between black patients and white patients. The cumulative propensity score discriminated race, with a C statistic of 0.89, and the propensity scores were associated with 1- and 5-year mortality rates (hazard ratio for the 75th percentile of the propensity score vs 25th percentile, 1.72; 95% CI, 1.43-2.08; *P* < .001). Patients in the lowest propensity score quintile associated with being a black individual (regardless of whether they were of white or black race) had a 5-year mortality rate of 15.5%, while those in the highest quintile had a 5-year mortality rate of 31.0% (*P* < .001). After adjusting for the propensity associated with being a black patient, there was no significant mortality rate difference by race (adjusted hazard ratio, 1.09; 95% CI, 0.93-1.26; *P* = .37) and no statistical interaction between race and propensity score (*P* = .42).

**Conclusions and Relevance:**

Characteristics of black patients and white patients differed significantly at the time of admission for AMI. Those characteristics were associated with an approximately 3-fold difference in 5-year mortality rate following AMI and mediated most of the observed mortality rate difference between the races.

## Key Points

**Question:**

Does race serve as a surrogate for socioeconomic and clinical factors, and, after adjusting for those factors, do long-term mortality rates differ between black patients and white patients following acute myocardial infarction?

**Findings:**

In this cohort study of 6402 patients from 2 acute myocardial infarction registries, self-identified black patients and white patients differed in several clinical and socioeconomic characteristics. The higher the prevalence of characteristics associated with being a black patient, the higher the 5-year mortality rate, but no differences were observed between black patients and white patients with similar characteristics.

**Meaning:**

A greater prevalence of characteristics associated with black race, but not race itself, was associated with higher mortality risk after acute myocardial infarction.

## Teaser

This cohort study uses data from 2 US myocardial infarction registries to assess whether demographic or clinical characteristics differ between self-identified black patients and white patients or are associated with 1- or 5-year survival after acute myocardial infarction.

## Introduction

Disparities in cardiovascular care for racial and ethnic minorities in the United States have been well documented.^1,2,3,4,5^ For the care of patients with acute myocardial infarction (AMI), published data have shown that black patients are less likely to receive guideline-concordant care before an AMI^6^ or coronary revascularization after presentation^1,7^ and are at higher risk for adverse outcomes, including recurrent myocardial infarction (MI), rehospitalization, and, in most studies, death.^8^ Prior studies on racial disparities in cardiovascular care have largely focused on differences in treatment between black patients and white patients as opposed to other factors that may be associated with differences in outcomes. Thus, current public policy has focused on equalizing treatment between black patients and nonblack patients, with various initiatives targeting guidelines, protocols, and tools to reduce racial variations in treatment.^6^ Disparities in some cardiovascular process measures, and even outcomes, have improved,^9^ with strong protocol-driven processes of care appearing to reduce racial disparities in care and outcomes.^10^ Despite the publication of these strategies, inequalities in morbidity and mortality rates between black patients and white patients still persist following AMI.^10,11^

Recent studies have suggested that race may simply serve as a marker for myriad socioeconomic and health status characteristics that are associated with adverse outcomes, many of which are beyond the locus of control of individual health care professionals.^12,13,14^ However, recent editorials have explicitly called for more research to better illuminate what accounts for racial differences in outcomes, as a foundation for reducing disparities.^15^ Accordingly, a better understanding of patient characteristics associated with racial disparities in outcomes is needed.

Propensity scoring is routinely used in comparative effectiveness research to statistically balance the characteristics of patients treated with one strategy vs another. This allows for an estimation of the exposure’s effect by accounting for the covariates that predict exposure. This technique has also been used to look at racial differences in quality of life, rehospitalization, and related outcomes after MI.^16^ We sought to extend this work by using propensity scores to compare black patients and white patients across a range of patient characteristics, and to determine the extent to which racial differences in outcomes are associated with those factors that differ by race. Given the focus of the research community on differences in treatment, we also examined whether adjusting for treatment would eradicate disparities in outcomes associated with characteristics more prevalent in black patients. We conducted these analyses using 2 observational registries that prospectively collected detailed data on patients’ socioeconomic, health, social support, and psychological statuses, as well as their treatment, and examined how these characteristics differ by race, how they are associated with 1- or 5-year survival after AMI, and whether this association differed for black patients and white patients with similar characteristics.

## Methods

### Patient Population

We combined data from 2 prospective AMI registries, Prospective Registry Evaluating Myocardial Infarction: Events and Recovery (PREMIER) and Translational Research Investigating Underlying Disparities in Acute Myocardial Infarction Patients’ Health Status (TRIUMPH), which have been previously described.^17,18^ The PREMIER study enrolled 2498 patients from 19 hospitals from 2003 to 2004, and the TRIUMPH study enrolled 4340 patients from 24 hospitals from 2005 to 2008 (12 of the 24 TRIUMPH hospitals also participated in PREMIER). Both studies included patients who were 18 years or older and were hospitalized with an AMI confirmed by biochemical evidence of myocardial necrosis (elevated cardiac biomarkers) and either prolonged (>20 minutes) symptoms of myocardial ischemia or diagnostic electrocardiographic changes. This study followed the Strengthening the Reporting of Observational Studies in Epidemiology (STROBE) reporting guideline. The present study was approved by the institutional review boards of Saint Luke’s Hospital, Kansas City, Missouri, and the enrolling institutions. All patients gave written informed consent prior to participating. We limited our analyses to self-identified black patients and white patients, excluding patients of other races, including multiple (n = 409) or unknown (n = 27) race. The total population included 6402 patients from 31 centers.

### Data Collection

Data were prospectively collected from patient records reviews and from interviews conducted during the index admission. The record reviews captured patient comorbidities, clinical presentation, and in-hospital treatments. The interviews were conducted by trained study coordinators and captured patients’ self-identified race and detailed information about their health status, socioeconomic status, lifestyle habits, and psychosocial status. For both the PREMIER and TRIUMPH registries, patients were asked to describe their race and could select multiple racial groups. Survival through 1 and 5 years was assessed through queries of the National Death Index (Centers for Disease Control and Prevention).

### Statistical Analysis

Patient characteristics were categorized into thematic domains, within which each characteristic was compared between black patients and white patients using *t* tests for continuous variables and χ^2^ tests for categorical variables. The 8 domains and their individual components are provided in Table 1 and included demographic characteristics (age and sex), socioeconomic status (zip code, median income, educational level, work status, insurance, medication insurance, monthly financial reserves, economic burden of medical costs, and avoidance of care or not taking medication because of cost), social support (marital status, living alone, and Enhancing Recovery in Coronary Heart Disease social support score),^19,20^ lifestyle factors (smoking status, history of cocaine use, and body mass index calculated as weight in kilograms divided by height in meters squared), medical history (hyperlipidemia, hypertension, diabetes, prior MI, prior percutaneous coronary intervention, prior coronary artery bypass graft surgery, prior stroke or transient ischemic attack, chronic heart failure, coronary artery bypass graft left ventricular systolic function, chronic kidney disease, dialysis, chronic lung disease, and cancer), clinical presentation (ST-elevation MI, cardiac arrest, and initial hemoglobin), health status (Seattle Angina Questionnaire; 12-item Short-Form Health Survey [SF-12] physical and mental component summaries), and depressive symptoms (9-item Patient Health Questionnaire).

**Table 1.** TRIUMPH and PREMIER Patient Characteristics

Characteristic	White Patients (n = 4754)	Black Patients (n = 1648)	*P* Value^a^	Standardized Difference, %
Demographic				
Age, mean (SD), y	60.9 (12.5)	57.3 (12.5)	<.001	28.6
Sex, No. (%)				
Male	3367 (70.8)	908 (55.1)	<.001	33.0
Female	1387 (29.2)	740 (44.9)		
Geographic region, No. (%)				
Northeast	1020 (21.5)	114 (6.9)	<.001	75.8
South	1278 (26.9)	985 (59.8)		
Midwest	2049 (43.1)	505 (30.6)		
West	407 (8.6)	44 (2.7)		
Socioeconomic status				
Educational level, No. (%)				
Did not complete high school	736 (15.6)	541 (33.3)	<.001	57.5
Completed high school	1377 (29.2)	550 (33.9)		
Some college or vocational school	1414 (30.0)	390 (24.0)		
Graduated from college	743 (15.8)	93 (5.7)		
Postgraduate degree	441 (9.4)	50 (3.1)		
Work status, No. (%)				
Full-time	1989 (42.2)	401 (24.7)	<.001	38.8
Part-time	430 (9.1)	148 (9.1)		
Not working	2293 (48.7)	1074 (66.2)		
Health insurance, No. (%)	3978 (86.1)	1129 (71.2)	<.001	36.9
Insurance coverage for medications, No. (%)	3693 (79.1)	980 (60.6)	<.001	41.1
Monthly financial situation, No. (%)				
Some money left over	2515 (54.4)	388 (24.2)	<.001	68.5
Just enough to make ends meet	1485 (32.1)	699 (43.6)		
Not enough to make ends meet	624 (13.5)	517 (32.2)		
Medical costs have been an economic burden, No. (%)				
Severe	442 (9.4)	205 (12.7)	<.001	19.7
Moderate	421 (9.0)	168 (10.4)		
Somewhat	455 (9.7)	224 (13.9)		
A little	441 (9.4)	139 (8.6)		
Not at all	2926 (62.5)	877 (54.4)		
Avoided obtaining health care because of cost, No. (%)	976 (20.9)	442 (27.5)	<.001	15.4
Medication not taken because of cost, No. (%)				
Always	99 (2.1)	63 (3.9)	<.001	31.8
Frequently	187 (4.0)	121 (7.5)		
Occasionally	299 (6.4)	197 (12.2)		
Rarely	271 (5.8)	128 (7.9)		
Never	3849 (81.8)	1110 (68.6)		
Zip code median income, mean (SD), $	56 089.3 (21 707.3)	37 815.4 (13 555.7)	<.001	101.0
Zip code median income, No. (%)				
2500 to <25 000	105 (2.2)	321 (19.8)	<.001	99.9
25 000 to <50 000	2120 (45.4)	1052 (65.0)		
50 000 to <75 000	1620 (34.7)	214 (13.2)		
75 000 to <100 000	606 (13.0)	30 (1.9)		
100 000 to 250 000	216 (4.6)	1 (0.1)		
Social factors	105 (2.2)	321 (19.8)		
Marital status, No. (%)				
Married	2996 (63.4)	544 (33.4)	<.001	66.9
Divorced or separated	837 (17.7)	474 (29.1)		
Widowed	524 (11.1)	230 (14.1)		
Single	372 (7.9)	381 (23.4)		
Lives alone	1027 (21.9)	472 (29.1)	<.001	16.7
ENRICHD social support score	22.2 (4.2)	21.3 (5.1)	<.001	19.5
Lifestyle factors				
Smoking status, No. (%)				
Current	1634 (34.6)	712 (43.7)	<.001	27.2
Former	1785 (37.8)	415 (25.5)		
Never (or <100 total)	1303 (27.6)	503 (30.9)		
History of cocaine use, No. (%)	125 (2.6)	229 (13.9)	<.001	41.8
BMI, mean (SD)	29.4 (6.0)	29.9 (7.3)	.002	8.5
Cardiac history, No. (%)				
MI	937 (19.7)	408 (24.8)	<.001	12.2
CABG	622 (13.1)	144 (8.7)	<.001	14.0
PCI	921 (19.4)	285 (17.3)	.06	5.4
CVA	214 (4.5)	134 (8.1)	<.001	15.0
TIA	129 (2.7)	34 (2.1)	.15	4.3
Chronic heart failure	319 (6.7)	315 (19.1)	<.001	37.6
LV systolic function				
Normal	2752 (58.0)	1000 (60.8)	<.001	27.6
Mild	1039 (21.9)	252 (15.3)		
Moderate	645 (13.6)	177 (10.8)		
Severe	312 (6.6)	216 (13.1)		
Noncardiac history, No. (%)				
Hypercholesterolemia	2426 (51.0)	697 (42.3)	<.001	17.6
Hypertension	2904 (61.1)	1303 (79.1)	<.001	40.0
Diabetes	1235 (26.0)	667 (40.5)	<.001	31.1
Chronic renal failure	252 (5.3)	289 (17.5)	<.001	39.2
Dialysis	38 (0.8)	87 (5.3)	<.001	26.3
Chronic lung disease	452 (9.5)	170 (10.3)	.34	2.7
Cancer (other than skin)	409 (8.6)	92 (5.6)	<.001	11.8
Presentation				
ST-elevation MI, No. (%)	2301 (48.4)	458 (27.8)	<.001	43.4
Cardiac arrest, No. (%)	195 (4.1)	29 (1.8)	<.001	13.9
Hemoglobin, mean (SD), g/dL	14.1 (2.1)	13.0 (2.2)	<.001	47.6
Health status, mean (SD)				
SAQ summary score	78.6 (17.6)	73.0 (20.5)	<.001	29.0
SAQ physical limitation score	87.7 (20.5)	76.7 (28.0)	<.001	44.9
SAQ angina stability score	41.9 (22.7)	43.8 (23.3)	.004	8.2
SAQ angina frequency score	85.6 (21.0)	83.8 (22.0)	.002	8.5
SAQ quality of life score	64.5 (22.8)	58.8 (25.3)	<.001	23.5
SF-12 physical component summary	43.3 (12.3)	39.7 (12.5)	<.001	28.5
SF-12 mental component summary	50.2 (11.1)	47.9 (12.4)	<.001	19.4
Depression, mean (SD)				
PHQ-9 depression score	5.3 (5.3)	5.4 (5.5)	.83	0.6
In-hospital treatment, No. (%)				
Diagnostic catheterization	4463 (93.9)	1294 (78.5)	<.001	45.7
Revascularization	3785 (79.6)	848 (51.5)	<.001	62.0
Primary PCI	2074 (43.6)	386 (23.4)	<.001	43.8
Aspirin (DC)	4463 (93.9)	1480 (89.8)	<.001	14.9
β-Blocker (DC)	4277 (90.0)	1397 (84.8)	<.001	15.7
ACE inhibitor or ARB (DC)	3471 (73.0)	1229 (74.6)	.22	3.6
Statin (DC)	4093 (86.1)	1334 (80.9)	<.001	13.9
Patient instructions				
Cardiac rehabilitation	2346 (49.3)	381 (23.1)	<.001	56.7
Smoking cessation	1701 (35.8)	561 (34.0)	.20	3.6

Abbreviations: ACE, angiotensin-converting enzyme; ARB, angiotensin II receptor blocker; BMI, body mass index (calculated as weight in kilograms divided by height in meters squared); CABG, coronary artery bypass graft; CVA, cerebral vascular accident; DC, at discharge; ENRICHD, Enhancing Recovery in Coronary Heart Disease; LV, left ventricular; MI, myocardial infarction; PCI, percutaneous coronary intervention; PHQ-9, 9-item Patient Health Questionnaire; PREMIER, Prospective Registry Evaluating Myocardial Infarction: Events and Recovery; SAQ, Seattle Angina Questionnaire; SF-12, 12-item Short-Form Health Survey; TIA, transient ischemic attack; TRIUMPH, Translational Research Investigating Underlying Disparities in Acute Myocardial Infarction Patients’ Health Status.

SI conversion factor: To convert hemoglobin to grams per liter, multiply by 10.0.

^a^ Continuous variables compared using *t* tests, and categorical variables compared using χ^2^ or Fisher exact tests.

To assess how the distributions of the 8 domains varied by race, we constructed multiple propensity scores for being a black individual using patients’ self-identified race as the outcome. Initially, we created 8 scores based on each domain individually to identify which domains most discriminated the 2 races. We then created 8 more scores by sequentially introducing all domains, 1 at a time in the aforementioned order, to demonstrate the cumulative contributions of these domains to discriminating race. Propensity scores were calculated using logistic regression, with race as the dependent variable and each of the relevant domain variables as independent variables. Nonlinear effects for continuous variables were modeled using restricted cubic splines. The potential for overfitting as evaluated by bootstrap validation of the full model calibration slope was 0.99, and compared with a perfect calibration slope of 1.0, this score indicated minimal overfitting risk. We compared propensity scores between race groups graphically, using smoothed kernel density estimates of the propensity score distributions, and quantitatively using the C statistic, where higher C statistic values indicated that the included factors more strongly discriminated race. Finally, using the final propensity score including all covariates, we estimated the association of the propensity to be a black individual with 1- and 5-year all-cause mortality using Cox regression models. The models included fixed effects for race, the propensity score, a propensity-by-race interaction, and a random effect for site to account for clustering of observations. The propensity score effect was estimated using 4-knot-restricted cubic splines to allow for nonlinear trends. Proportional hazards assumptions were tested by Schoenfeld residuals and were found to be satisfied in all cases (*P* > .20 for testing departures from proportionality). This latter analysis not only described the risk of mortality as a function of a greater prevalence of characteristics associated with being a black individual, but also compared whether these associations with mortality risk differed between black patients and white patients. To highlight the differences in 5-year mortality rate as a function of having characteristics associated with being a black individual, we estimated the hazard ratio (HR) associated with being at the 75th percentile of the propensity score vs at the 25th percentile.

Overall, 1697 of 6402 patients (26.5%) were missing data on at least 1 propensity score covariate, and this rate was higher in black patients (558 of 1648 [33.9%] vs 1139 of 4754 [24.0%], *P* < .001). The most common missing items were body mass index (344 of 6402 [5.4%] overall; 215 of 1648 [13.0%] for black patients vs 129 of 4754 [2.7%] for white patients; *P* < .001) and SF-12 health status scores (292 of 6402 [4.6%], *P* = .28 by race)and 9-item Patient Health Questionnaire depression scores (373 of 6402 [5.8%]; *P* = .63 by race). Missing values of covariates were imputed using multiple imputation by chained equations incorporating race, all observed covariates, and outcomes.^21^ The missing rates for propensity score covariates were tabulated and are given in the eTable in the Supplement. The 5-year survival status was complete for all but 2 patients.

Because treatments are known to vary by race and evidence demonstrates differences in survival among hospitals treating larger proportions of black patients,^22,23^ we explored, as a secondary analysis, whether further adjustment for treatment and site would alter the association between the propensity to be a black patient and survival. This analysis could also estimate whether equalizing treatment might eliminate racial differences in outcomes.

A 2-sided *P* < .05 denoted statistical significance. All analyses were conducted from December 2016 to July 2018 with SAS, version 9.4 (SAS Institute Inc) and R, version 3.4.4 (R Foundation for Statistical Computing).

## Results

Among the 6402 participants, 1648 (25.7%) were black, 2127 (33.2%) women, and the mean (SD) age was 60 (13) years. Black patients and white patients differed substantially in almost all demographic, socioeconomic, psychosocial, clinical, disease severity, and health status characteristics (Table 1). For example, the mean (SD) age of black patients was 57 (12) years, whereas the mean (SD) age of white patients was 61 (12) years (*P* < .001), and 908 black patients (55%) were male compared with 3367 white patients (71%). Of the characteristics that were more common among black patients, although some favored survival (eg, younger age and less likely to present with cardiac arrest), most were known to be associated with worse survival, including lower socioeconomic status, poorer social support, greater history of MI and heart failure, and worse health status.

### Distribution of Propensity Scores by Race

**Figure 1.**
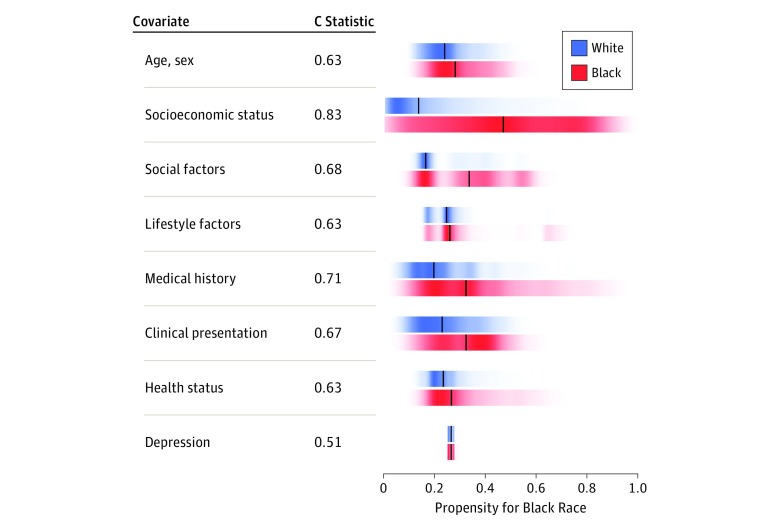
Color-Gradient Density Plots Indicating the Propensity for Being a Black Individual Based on Each Individual Domain, Analyzed Separately for White Patients and for Black Patients Color intensity reflects concentration of data; black lines indicate median propensity scores; and overlap of scores in a domain for white patients and black patients indicates that for that domain, the patients are more similar.

Figure 1 shows color-gradient density plots of the propensity scores for being a black individual, separately for white patients and black patients, based on each of the 8 domains of patient characteristics. The greatest separation between the 2 races was observed for socioeconomic factors. Based on the 8 socioeconomic status factors, black patients had a median propensity score of being a black individual of 48.2%, with the first quartile at 27.4%. By contrast, the median propensity score among white patients was 11.7%, with the third quartile at 25.3%. The next most distinguishing characteristics were social factors, followed by medical history.

**Figure 2.**
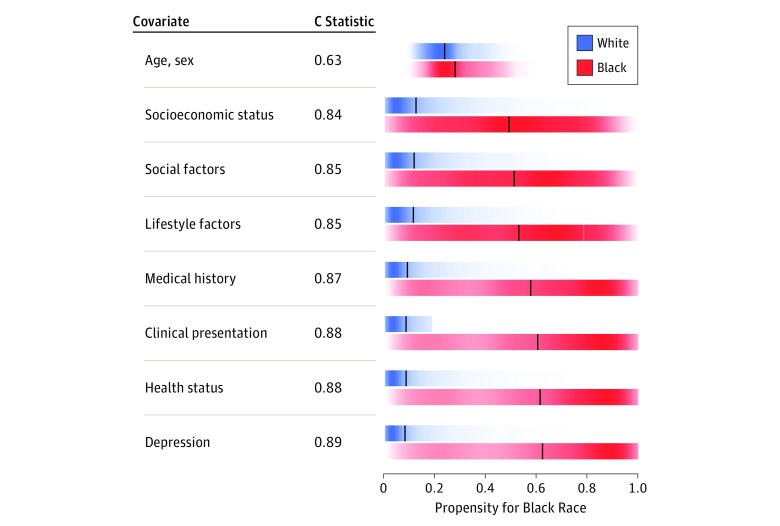
Color-Gradient Density Plots Indicating the Propensity for Being a Black Individual Based on the Listed Domain and All Prior Domains (Each Step Added a Domain), Analyzed Separately for White Patients and Black Patients Color intensity reflects concentration of data; black lines indicate median propensity scores; and overlap of scores for white patients and black patients indicates similarity of patients.

In cumulative logistic regression models of patient characteristics associated with being a black individual (Figure 2), we found substantial overlap between white patients and black patients when only age and sex were included. However, after sequentially including each of the additional clusters, a progressively larger separation was observed, indicating less and less overlap of patient characteristics. The C statistic for the final model was 0.89, indicating strong discriminatory power to determine the race of the patient based only on the nonrace/nonethnic patient characteristics present on admission. Table 2 provides a summary of the independent strengths of association of each of the propensity score covariates with race based on the final propensity score logistic regression model. Most notably, the largest contributing factor, by far, was the median income of the patient’s zip code.

**Table 2.** Independent Strengths of Association of Each of the Propensity Score Covariates With Race, From the Final Propensity Score Logistic Regression Model

Covariate	Wald χ^2^
Age	51.7
Sex	5.5
Educational level	21.9
Working status	7.1
Health insurance	5.3
Insurance coverage for medication	6.5
Monthly financial situation	43.4
Medical costs an economic burden	12.1
Health care not obtained because of cost	17.6
Medication not taken because of cost	7.7
Zip code median income	383.5
Marital status	60.4
Lived alone	0.3
ENRICHD social support score	1.9
Smoking status	12.1
History of cocaine use	29.0
BMI	1.9
Prior MI	3.1
Prior CABG	17.2
Prior PCI	7.9
Prior CVA	3.9
Prior TIA	0.9
Chronic heart failure	11.8
LV systolic function	15.4
Hypercholesterolemia	15.3
Hypertension	52.6
Diabetes	3.3
Chronic renal failure	20.2
Dialysis	2.2
Chronic lung disease	4.8
Cancer (other than skin)	3.7
ST-elevation MI	30.4
Cardiac arrest	3.0
Hemoglobin (g/dL)	89.4
SAQ physical limitation score	24.5
SAQ angina stability score	11.8
SAQ angina frequency score	6.8
SAQ quality of life score	3.4
SF-12 physical component summary	1.4
SF-12 mental component summary	5.0
PHQ-9 depression score	44.7

Abbreviations: BMI, body mass index (calculated as weight in kilograms divided by height in meters squared); CABG, coronary artery bypass graft; CVA, cerebral vascular accident; ENRICHD, Enhancing Recovery in Coronary Heart Disease; LV, left ventricular; MI, myocardial infarction; PCI, percutaneous coronary intervention; PHQ-9, 9-item Patient Health Questionnaire; SAQ, Seattle Angina Questionnaire; SF-12, 12-item Short-Form Health Survey; TIA, transient ischemic attack.

### Association Between the Propensity to Be a Black Individual and Mortality Rate

**Figure 3.**
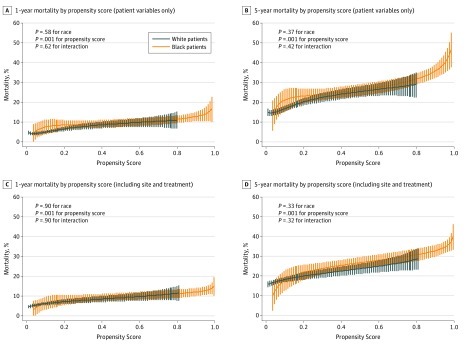
Association Between the Propensity to Be a Black Individual and 1- and 5-Year Mortality Rates *P* values for (race × propensity) interaction scores.

Overall, the 1-year mortality rate was 10.6% (174 of 1648) for black patients compared with 5.8% (275 of 4754) for white patients, and the 5-year mortality rate was 28.9% (476 of 1648) for black patients compared with 18.0% (856 of 4754) for white patients. The unadjusted 5-year mortality HR for black vs white race was 1.72 (95% CI, 1.54-1.92; *P* < .001). There was a strong association between the propensity associated with being a black individual and increased risk of mortality, regardless of patient race (Figure 3). Using the full propensity score, the 1-year mortality rate ranged from approximately 5% among those with the lowest prevalence of characteristics associated with being a black individual to approximately 12% for those with the highest prevalence. Similarly, the 5-year probability of mortality ranged from roughly 15% to 40%. Patients in the lowest propensity score quintile associated with being a black individual (regardless of whether they were of white or black race) had a 5-year mortality rate of 15.5%, while those in the highest quintile had a 5-year mortality rate of 31.0% (*P* < .001). The HR for the 75th percentile of the propensity score, as compared with the 25th percentile, was 1.72 (95% CI, 1.43-2.08; *P* < .001). There was no significant difference in mortality risk between black patients and white patients after adjusting for the propensity score (adjusted HR, 1.09; 95% CI, 0.93-1.26; *P* = .37) and no statistical interaction between race and propensity score (*P* = .42). These data suggested that race was a marker for myriad factors that were strongly associated with mortality rate and that there was no residual association between race and mortality rate after accounting for other demographic, socioeconomic, psychosocial, clinical, and health status factors. The mediation proportion, (unadjusted HR − adjusted HR)/(unadjusted HR − 1), was 91.7%, suggesting that patient factors explained approximately 92% of the crude difference in mortality risk between black patients and white patients.

In secondary analyses of 5-year mortality rate, site of care and in-hospital treatment were added to the propensity score. When site of care was added to the propensity score, the HR decreased slightly from 1.72 to 1.66 (95% CI, 1.37-2.01; *P* < .001). After further including treatment received (primary percutaneous coronary intervention, revascularization, aspirin, β-blockers, angiotensin-converting enzyme inhibitors, or angiotensin II receptor blockers at discharge as well as smoking cessation and cardiac rehabilitation referral), the HR comparing the 75th and 25th percentiles of propensity scores remained virtually unchanged at 1.66 (95% CI, 1.37-2.01; *P* < .001), suggesting that even after accounting for both site of care and treatment, there was a significant association with having more characteristics associated with being a black individual and the 5-year mortality rate. When comparing the models with or without treatment in the model, we found that the global *P* value for adding treatment to the model was .21, suggesting that treatment differences by race did not significantly alter the association of our primary analysis.

## Discussion

Eradicating racial disparities in survival after AMI is a national priority,^24^ but, to date, most efforts to understand racial differences in outcomes have focused on differences in treatment during the AMI hospitalization.^24,25^ Although treatment differences are important, they may not account for all of the observed racial disparities in outcomes. In the present study, we found that black patients and white patients differed markedly across a range of prognostically important characteristics and that, after accounting for the characteristics associated with being a black patient, there were no differences in long-term survival between self-reported black patients and white patients. This suggests that race is a marker of many important risk factors that are associated with mortality. Although not definitive, these findings indicate that, even without controlling for genetic factors, the mortality risk after AMI is not different between black patient and white patients after adjusting for socioeconomic, psychosocial, and health status characteristics.

These analyses extend prior data showing that black patients with AMI may have worse long-term outcomes than white patients and that these differences did not persist after adjusting for patient factors and site of care.^16^ In addition, our findings provide a different perspective to the extensive literature on racial disparities in survival after AMI. There has been a wealth of data on differences in treatment, discharge measures, and other quality of care indicators and the contribution of those differences to outcomes between black patients and white patients;^12,14,16^ however, we primarily focused on prehospital characteristics and showed an almost 3-fold increase in 5-year mortality risk across the range of attributes associated with being a black individual. Even after controlling for site of care and treatment, there was a significant correlation with the propensity of the characteristics associated with being a black individual and survival. Collectively, characteristics mediated approximately 90% of the observed mortality rate difference between races.

Some prior studies have addressed the myriad differences between black patients and white patients by developing or examining race-specific models,^26^ but many have not documented the characteristics that differ between black patients and white patients that support the use of such models. We found that socioeconomic and social factors were 2 of the most important factors in differentiating white patients and black patients, and because these are seldom incorporated into risk models, this may explain why race-specific models may be more accurate than an overall model that includes race. In addition, of all the propensity score covariates examined in the present study, the median income of the patient zip code was the strongest contributor. This strengthens the finding in other studies that have shown, for example, that black patients who live in neighborhoods with higher segregation scores (indices that measure the degree to which the minority group is distributed differently than white individuals across census tracts) have also been associated with higher cardiovascular disease incidence, even after adjustments for individual-level demographic characteristics or traditional cardiovascular disease risk factors.^27^ Our finding that socioeconomic status–related variables were the strongest differentiator between black patients with AMI and white patients with AMI suggests that further understanding of the mechanism by which socioeconomic status affects survival may be an important target for future research.

### Limitations

The present findings should be interpreted in the context of several potential limitations. First, although the TRIUMPH and PREMIER registries included data from hospitals with good geographic representation across the United States, the data may not be generalizable throughout the country. Second, because this was an observational study, there may be other important characteristics that differ by race that were not included in our models. Further research is needed to identify those factors that may both differ substantially by race and be associated with outcome so that additional targets for intervention can be identified. Third, the registries relied on self-identified racial categories and did not include genomic data; thus, contributions of the genetic components of race to outcomes could not be determined. African ancestry was not accounted for in this study, and future studies would benefit from collecting genetic and ancestral data. Fourth, the registries were created more than a decade ago. Treatments and outcomes may have changed with time, but there is no reason to believe that the association with the propensity associated with being a black individual and outcomes would have changed, although the absolute rates of death may have diminished. Given the extensive patient-centered data collected in the PREMIER and TRIUMPH registries, these were the best data from which to explore our hypothesis, but replication in a more contemporary population that collects the same detailed patient-centered data would be important to show that these associations have not changed with time.

## Conclusions

We aimed to determine the degree to which race served as a proxy for differences in survival after AMI. We derived a model that showed a marked difference in mortality rate based on characteristics that were more prevalent in black individuals, but we found no differences in 1- and 5-year survival rates between black patients and white patients with similar characteristics. Our data suggest that there are myriad characteristics associated with race that likely contribute to racial disparities in AMI outcomes. More compelling is that those factors were strongly associated with mortality, and this finding should prompt new research into novel treatment strategies that can address novel potential mediators of racial disparities in survival after AMI.

## Notes

Supplement. **eTable.** TRIUMPH/PREMIER Racial Disparities Propensity Score Covariate Missingness by Race
