# Supplementary material for: Racial Disparities in Patient Characteristics and Survival After Acute Myocardial Infarction
Source: JAMA Netw Open. 2018 Nov 2;1(7):e184240. doi: 10.1001/jamanetworkopen.2018.4240 (PMC6324589; doi:10.1001/jamanetworkopen.2018.4240)
Supplement: Supplement. — eTable. TRIUMPH/PREMIER Racial Disparities Propensity Score Covariate Missingness by Race [file jamanetwopen-1-e184240-s001.pdf]

# Supplementary Online Content

Graham GN, Jones PG, Chan PS, Arnold SV, Krumholz HM, Spertus JA. Racial disparities in patient characteristics and survival after acute myocardial infarction. *JAMA Netw Open*. 2018;1(7):e184240. doi:10.1001/jamanetworkopen.2018.4240

**eTable.** TRIUMPH/PREMIER Racial Disparities Propensity Score Covariate Missingness by Race

This supplementary material has been provided by the authors to give readers additional information about their work.

© 2018 Graham GN et al. *JAMA Network Open*.

**eTable.** TRIUMPH/PREMIER Racial Disparities Propensity Score Covariate Missingness by Race

| | n = 6402 | White/Caucasian<br>n = 4754 | Black/African-American<br>n = 1648 | P-Value |
|---|---|---|---|---|
| Any missing covariate | 26.5% | 24.0% | 33.9% | < 0.001 |
| *Demographics* | | | | |
| Age | 0.0% | 0.0% | 0.0% | 1.000 |
| Sex | 0.0% | 0.0% | 0.0% | 1.000 |
| *Socioeconomic status* | | | | |
| Education | 1.0% | 0.9% | 1.5% | 0.057 |
| Working status | 1.0% | 0.9% | 1.5% | 0.029 |
| Health insurance | 3.1% | 2.8% | 3.8% | 0.037 |
| Insurance coverage for medications | 1.8% | 1.8% | 1.9% | 0.687 |
| Monthly financial situation | 2.7% | 2.7% | 2.7% | 0.889 |
| Medical costs have been an economic burden | 1.6% | 1.5% | 2.1% | 0.062 |
| Avoided getting health care due to cost | 2.0% | 1.9% | 2.5% | 0.126 |
| Not taken medication due to cost | 1.2% | 1.0% | 1.8% | 0.020 |
| ZIP code median income | 1.8% | 1.8% | 1.8% | 0.979 |
| *Social factors* | | | | |
| Marital status | 0.7% | 0.5% | 1.2% | 0.007 |
| Lives alone | 1.3% | 1.2% | 1.6% | 0.134 |
| ENRICHD Social Support Score | 2.9% | 2.9% | 2.9% | 0.846 |
| *Lifestyle factors* | | | | |
| Smoking status | 0.8% | 0.7% | 1.1% | 0.095 |
| History of cocaine use | 0.0% | 0.0% | 0.0% | 1.000 |
| BMI | 5.4% | 2.7% | 13.0% | < 0.001 |
| *Cardiac history* | | | | |
| Prior MI | 0.0% | 0.0% | 0.0% | 1.000 |
| Prior CABG | 0.0% | 0.0% | 0.0% | 1.000 |
| Prior PCI | 0.0% | 0.0% | 0.0% | 1.000 |
| Prior CVA | 0.0% | 0.0% | 0.0% | 1.000 |
| Prior TIA | 0.0% | 0.0% | 0.0% | 1.000 |
| Chronic heart failure | 0.0% | 0.0% | 0.0% | 1.000 |
| LV systolic function | 0.1% | 0.1% | 0.2% | 0.702 |
| *Non-cardiac history* | | | | |
| Hypercholesterolemia | 0.0% | 0.0% | 0.0% | 1.000 |

© 2018 Graham GN et al. *JAMA Network Open*.

| | n = 6402 | White/Caucasian<br>n = 4754 | Black/African-American<br>n = 1648 | P-Value |
|---|---|---|---|---|
| Hypertension | 0.0% | 0.0% | 0.0% | 1.000 |
| Diabetes | 0.0% | 0.0% | 0.0% | 1.000 |
| Chronic renal failure | 0.0% | 0.0% | 0.0% | 1.000 |
| Dialysis | 0.0% | 0.0% | 0.0% | 1.000 |
| Chronic lung disease | 0.0% | 0.0% | 0.0% | 1.000 |
| Cancer (other than skin) | 0.0% | 0.0% | 0.0% | 1.000 |
| *Presentation* | | | | |
| ST-elevation MI | 0.0% | 0.0% | 0.0% | 1.000 |
| Cardiac arrest | 0.5% | 0.3% | 0.9% | 0.001 |
| Hemoglobin (g/dL) | 0.2% | 0.1% | 0.2% | 0.724 |
| *Health status* | | | | |
| SAQ Physical Limitation Score | 0.3% | 0.3% | 0.4% | 0.662 |
| SAQ Angina Stability Score | 1.8% | 1.8% | 1.8% | 0.931 |
| SAQ Angina Frequency Score | 0.4% | 0.3% | 0.5% | 0.239 |
| SAQ Quality of Life Score | 0.6% | 0.5% | 0.8% | 0.091 |
| SF-12 Physical Component Summary | 4.6% | 4.4% | 5.0% | 0.283 |
| SF-12 Mental Component Summary | 4.6% | 4.4% | 5.0% | 0.283 |
| *Depression* | | | | |
| PHQ-9 Depression Score | 5.8% | 5.7% | 6.1% | 0.626 |

*Continuous variables compared using Student's T-test.*
*Categorical variables compared using chi-square or Fisher's exact test.*

© 2018 Graham GN et al. *JAMA Network Open*.
